# Dual Acridine/Thiophenol Photocatalysis for the Production of Advanced Drop-in Biofuels

**DOI:** 10.1021/acsomega.5c06908

**Published:** 2025-09-25

**Authors:** Jhudson Guilherme Leandro de Araujo, Jannyely Moreira Neri, Aruzza Mabel de Morais Araújo, Carlos Alberto Martínez-Huitle, Amanda Duarte Gondim, Lívia Nunes Cavalcanti

**Affiliations:** Federal University of Rio Grande do Norte, Institute of Chemistry, Campus Universitário, Lagoa Nova, Natal, RN 59072-970, Brazil

## Abstract

A practical and efficient dual photocatalytic system based on acridine and thiol has been developed for the production of advanced drop-in biofuels from vegetable and waste oils. This methodology enables the hydrodecarboxylation of fatty acids under mild conditions, metal- and hydrogen-free, using DCM as the solvent, which can be easily recovered and reused in the reaction. C_*n*–1_ hydrocarbons were obtained in yields exceeding 70% from isolated fatty acids, while complex hydrocarbon mixtures were produced with conversions of up to 99% from hydrolyzed fatty acids derived from soybean oil, palm oil, distillers corn oil, and used cooking oil. The resulting hydrocarbon fractions exhibit compositional profiles compatible with diesel and biojet fuel specifications, demonstrating their potential for direct application in commercial fuel blends. Compared with conventional thermochemical processes, this system offers a sustainable and economical alternative for converting waste feedstocks into second-generation biofuels.

## Graphical abstract

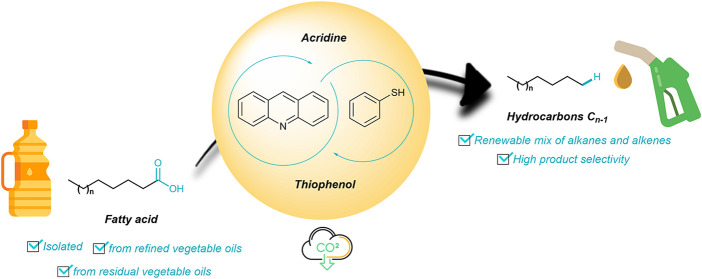

## 1 Introduction

The planet has already warmed by 1.5 °C since the preindustrial era, resulting in recurrent environmental consequences such as heat waves and irreparable flood damage.^1–6^ Based on these data, achieving the central goal of the Paris Agreement, to limit the Earth’s temperature increase to less than 2 °C above preindustrial levels, has become even more challenging. In this context, decarbonizing the transportation sector is considered a crucial strategy to mitigate greenhouse gas emissions and limit global warming.^7^

In 2022, approximately 36 GtCO_2_ were emitted by industrial processes and fossil fuel combustion. Of these emissions, 17.9% originated from land transportation, 3.1% from international bunkers (aviation and international maritime transport), and 0.9% from domestic aviation. Therefore, rapidly replacing petrochemical fuels with renewable alternatives is imperative.^7,8^

Biofuels such as biodiesel and bioethanol, are already part of the global energy matrix but present technical limitations due to their oxygenated structures. For example, biodiesel suffers from poor cold-flow properties, lower volumetric energy density, and low oxidative stability, preventing it from fully replacing fossil diesel.^9^ For similar reasons, it is unsuitable to replace aviation kerosene.^10^ Given these challenges, new technologies aimed at producing drop-in biofuels have gained prominence.

Among them, thermochemical routes have enabled significant advances in the conversion of biomass into hydrocarbons. However, these approaches typically require high temperatures and pressures, as well as hydrogen that is often derived from fossil sources—factors that lead to high energy consumption and operational costs, ultimately compromising the commercial competitiveness of the process.^11^

In this context, the photocatalytic hydrodecarboxylation of fatty acids has emerged as a promising alternative method for generating renewable hydrocarbons under ambient temperature and pressure (Figure 1a). Photoredox approaches have demonstrated high selectivity; however, despite these advances, the use of complex lipid feedstocks such as vegetable oils for producing drop-in biofuels still represents a gap to be explored.

**Figure 1:**
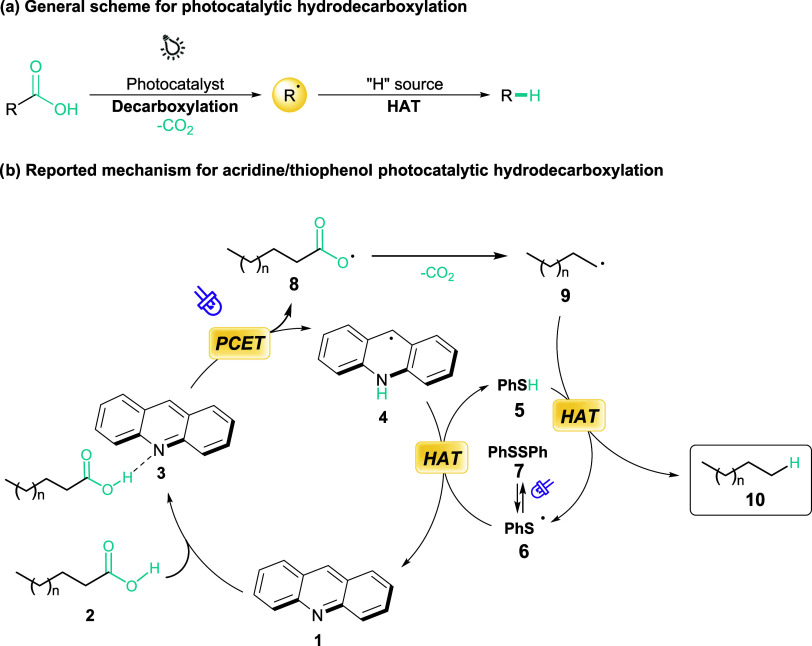
Scheme for photocatalytic hydrodecarboxylation.

For example, only the Wang group has reported an efficient synthesis of renewable hydrocarbons from residual biomass.^12^ Their method achieved the conversion of nonedible soybean fatty acids (SBFAs) and tall oil fatty acids (TOFAs)—byproducts of soybean oil refining and the pulp industry, respectively—into C_*n*–1_ hydrocarbons with conversions above 90%. However, the methodology relies on a Pt-TiO_2_ metal catalyst under ultraviolet (UV) light irradiation and requires a pressurized hydrogen atmosphere, which may limit its sustainability due to the use of precious metals and demanding reaction conditions.

In contrast, metal-free organophotoredox methods have emerged as more sustainable alternatives. Initially reported by Oda and collaborators in the late 20th century,^13^ these strategies gained momentum after 2015, when the Nicewicz group^14^ demonstrated hydrodecarboxylation of tridecanoic acid into *n*-dodecane under visible light, albeit with low yield. This system employed Fukuzumi’s acridinium photocatalyst and diphenyl disulfide as a cocatalyst for hydrogen atom transfer (HAT). Subsequently, Li and collaborators improved the method, achieving yields above 80% after 12 h, although the synthesis of the mesityl-substituted acridinium photocatalyst involves multiple steps and costly reagents.^15,16^

Recently, our group reported a dual photocatalytic system based on 9-(2-chlorophenyl)­acridine and thiophenol (PhSH), obtaining yields higher than 99% of renewable hydrocarbons from various fatty acids under mild conditions and without gaseous hydrogen.^17^ We also demonstrated, for the first time, direct and selective conversion of fatty acids from vegetable oil into hydrocarbon mixtures using an organophotoredox method. The proposed mechanism, based on previous literature, involves photocatalytic decarboxylation coupled with proton transfer, followed by quenching of alkyl radicals via hydrogen atom transfer from the substrate itself, strongly aligning with the principles of atom economy and green chemistry (Figure 1b). Despite these advances, our previous study was limited to using licuri oil as feedstock, a native Brazilian biomass with low availability and limited relevance for the energy sector.

Therefore, we aimed to investigate whether applying a similar organophotoredox method could represent a viable strategy for producing potential drop-in biofuels via hydrodecarboxylation of biomasses already established in the biofuel market, such as soybean oil (SO) and palm oil (PO).^18^ Additionally, we decided to expand our study to include widely available residual biomasses such as waste cooking oil (WCO) and corn distillers oil (DCO), which present high potential for generating advanced second-generation biofuels while mitigating concerns related to food chain competition.^19^

For this purpose, we selected a photocatalytic system based on acridine (Acr) and PhSH, previously demonstrated by our group to be highly selective, metal-free, and capable of operating under visible light irradiation at ambient temperature and pressure.^17,20^ Both catalysts are low-cost and commercially available (Section 6, Supporting Information), contributing to the practical and sustainable viability of the methodology.

In this work, we present a photocatalytic hydrodecarboxylation route for converting conventional and residual lipid biomasses into hydrocarbon blends suitable for drop-in biofuels. To the best of our knowledge, this is the first organophotoredox approach using commercially available reagents applied to the synthesis of advanced second-generation biofuels under mild and operationally simple conditions. The methodology provides a conceptually distinct synthetic route toward renewable hydrocarbon fuels, contributing to the development of sustainable fuel technologies.

## 2 Results and Discussion

We began our studies by optimizing the hydrodecarboxylation of lauric acid (**1a**) using Acr (10 mol %) as a photocatalyst, PhSH (10 mol %) as a HAT cocatalyst, and DCM as a solvent. The reaction mixture was purged with N_2_ and irradiated with a 9W LED light (395 nm) for 24 h, yielding n-undecane (**2a**) with a 16% yield (Table 1, entry 1).

**Table 1:**
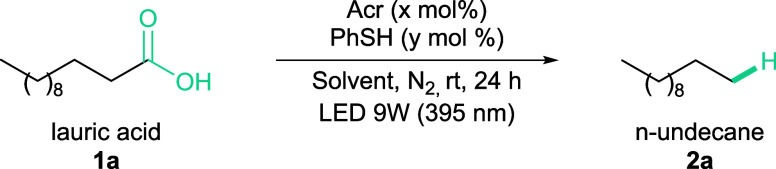
Optimization of the Reaction Conditions^b^

entry	*x*	*y*	solvent	yield (%)[Table-fn t1fn4]
1	10	10	DCM	16
2	10	10	MeCN	<5
3	10	10	toluene	19
4	10	10	AcOEt	23
5	10	10	DCE	8
6	10	10	MeOH	0
7	10	10	AcOEt/H_2_O (9:1)	15
8	10	10	DCM/H_2_O (9:1)	37
9	5	10	DCM/H_2_O (9:1)	<5
10	5	5	DCM/H_2_O (9:1)	<5
11	5	20	DCM/H_2_O (9:1)	17
12	10	20	DCM/H_2_O (9:1)	69
13	10	30	DCM/H_2_O (9:1)	62
14^c^	10	20	DCM/H_2_O (9:1)	72

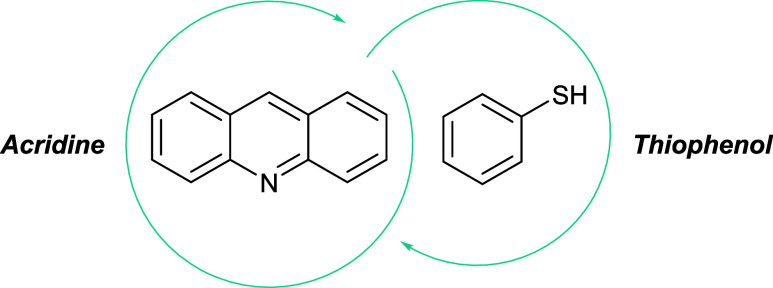

a Reaction performed on 0.4 mmol scale and 4.0 mL of solvent.

b 2.0 mL of solvent and LED 70W (405 nm) for 3 h.

c Determined by GC-FID using a calibration curve.

Extensive solvent screening (Table 1, entries 2–8 and Section 5.1, SI) revealed that DCM/H_2_O (9:1) was the optimal solvent for the reaction (Table 1, entry 7). In the next step, we investigated whether adding additives to the reaction could increase the product yield (Section 5.2, SI).

Initially, we tested the addition of bases, considering that carboxylate formation under similar conditions has been reported as a crucial step for decarboxylation to occur.^14–16,21,22^ We tested a range of organic and inorganic bases, but no significant improvement was observed. We then explored whether the addition of an oxidant could efficiently increase the yield of **2a**, following a previously effective strategy described by Larionov for acridine photocatalysis.^23,24^ We added 0.5 equiv of a series of oxidants, but none of the attempts promoted an increase in product formation. Additionally, we explored the addition of bromides and chlorides, known to form radical intermediates in photocatalytic reactions and facilitate hydrogen transfer via HAT process.^25^ However, once again, we did not observe an increase in the yield of **2a**.

Finally, we investigated the catalyst loading and the stoichiometric ratio between them. All attempts to reduce the catalyst loading to 5 mol % drastically decreased the reaction yield (Table 1, entries 9–11 and Section 5.3, SI). Therefore, we maintained a 10 mol % load of Acr and studied the influence of its stoichiometric ratio with the cocatalyst, PhSH. To our delight, when Acr and thiophenol were used in a 1:2 ratio, we obtained n-undecane with a 72% yield (Table 1, entry 12). We also found that increasing thiophenol to 30 mol % did not provide additional gain (Table 1, entry 13).

Next, we optimized the light source. When using a 7 W blue LED (≈450 nm), no product was formed. However, there is a time study (Section 5.5, SI) revealed the reaction is highly dependent on light intensity; use of a 70 W LED lamp (405 nm), whose emission is still well aligned with the acridine absorption range,^26^ reduced the reaction time to 3 h (Figure 2).

**Figure 2:**
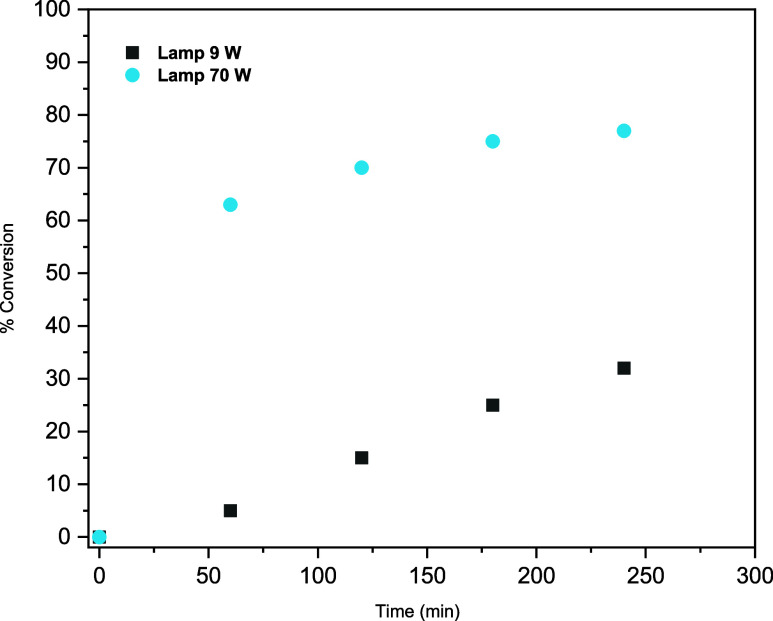
Comparison of the conversion of lauric acid under irradiation with a single 70 W LED lamp (405 nm) and a single 9 W LED lamp (395 nm).

Additionally, we determined that the reaction occurs without a loss of yield when the concentration of **1a** is increased to 0.2 M (Table 1, entry 14). Control experiments demonstrated that Acr, PhSH, and light are essential for this transformation, as no product was observed in the absence of any of these variables. The system also showed high tolerance to atmospheric oxygen, providing the desired hydrocarbon selectively under open-to-air conditions with minimal loss in yield (Section 5.6, SI), which highlights the method’s potential for practical biomass upgrading under ambient and practical conditions.

From these optimizations, the ideal conditions for hydrodecarboxylation of lauric acid (**1a**) to *n*-undecane (**2a**) were established as Acr (10 mol %), PhSH (20 mol %), and DCM/H_2_O (9:1, 0.2 M), under a N_2_ atmosphere.

With the optimized conditions in hand, we evaluated the efficiency of the methodology to produce C_*n*–1_ hydrocarbons from fatty acids with carbon chains ranging from C10 to C18 (Table 2). Product quantification was determined via GC-FID using an internal calibration curve (See SI).

**Table 2:**
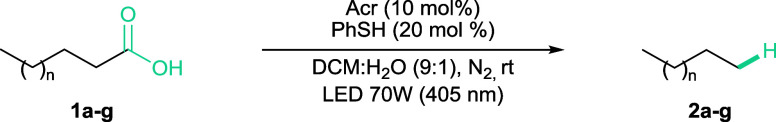

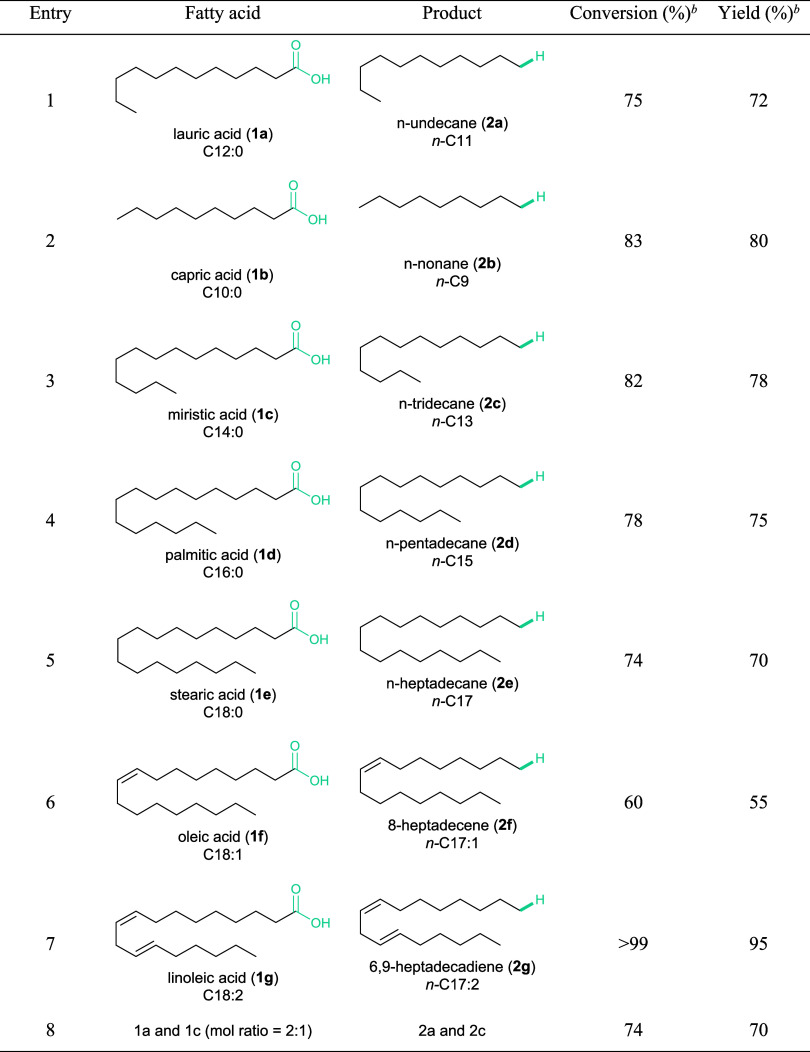
Scope of Fatty Acids for Photocatalytic Hydrodecarboxylation^a^

a Reaction conditions: fatty acid (0.4 mmol), Acr (10 mol %), PhSH (20 mol %), DCM/H_2_O (9:1, 2.0 mL), 70 W LED (405 nm), N_2_, rt, 3–24 h.

b Determined by GC-FID using a calibration curve.

Satisfactorily, we observed that all examined saturated fatty acids were converted to C_*n*–1_ alkanes with excellent selectivity and good yield (70–78%, Table 2, entries 1–5), including when a controlled mixture of lauric acid (**1a**) and myristic acid (1c) was tested (Table 2, entry 8). We also examined the hydrodecarboxylation of unsaturated fatty acids, which are substrates particularly challenging to convert compared to saturated fatty acids.^15,21,27,28^ We observed a lower yield of the corresponding C_*n*–1_ hydrocarbon for oleic acid (Table 2, entry 6), which may be due to steric and conformational effects associated with its internal double bond, limiting the efficiency of the hydrodecarboxylation. Interestingly, linoleic acid (1 g) afforded the C_*n*–1_ alkene (2 g) in quantitative conversion and 95% yield (Table 2, entry 7).

Encouraged by these results, we evaluated hydrodecarboxylation of real fatty acid mixtures from lipid biomass. Soybean oil (SO) and palm oil (PO), account for ≈60% of global lipid biomass used in biodiesel,^18^ as well as waste cooking oil (WCO) and distillers corn oil (DCO), industrial residues with no specific destination,^29,30^ were selected.^31–34^

SO and PO, primarily composed of linoleic acid (C18:2), is produced mainly in China, USA, and Brazil, with 62.3 million tonnes estimated global production in 2023, of which ≈15% is used for biodiesel.^19–21^ PO, representing over 40% of global vegetable oil supply, is mainly produced in Indonesia, Malaysia, and Thailand (≈79.3 million tonnes), with palmitic acid (C16:0) and oleic acid (C18:1) as main components.^20,22,23^

WCO, derived from frying oils, contains mainly oleic and palmitic acids and is produced at ≈16 million tonnes/year; improper disposal causes environmental issues.^30,35,36^ DCO, a byproduct of corn ethanol production, contains linoleic and oleic acids; with global corn harvest at 1.23 billion tonnes, ≈1.5 × 10^6^ m^3^ of DCO is recoverable from 56.8 × 10^6^ m^3^ of corn processing.^9,20,27^

The preliminary characterization of these biomasses showed a high content of unsaturated fatty acids (>60% in PO and WCO, ≈80% in SO and DCO) (Table 3), representing a known challenge for mild photocatalytic decarboxylation methods. Small differences between our data and the literature values may arise from variations in oil origin, cultivation conditions, or processing methods, although the overall fatty acid profile is similar.

**Table 3:** Fatty Acid Compositions of the Feedstocks

feedstock								
	soybean oil (SO)		palm oil (PO)		waste cooking oil (WCO)		distillery corn oil (DCO)	
	(wt%)							
fatty acid	Exp.^a^	Lit.^35^	Exp.^a^	Lit.^36^	Exp.^a^	Lit.^37^	Exp.^a^	Lit.^9^
palmitic acid (C16:0)	15.5	10.4	31.3	33.8	29.1	33.1	17.8	12.6
stearic acid (C18:0)	5.2	3.9	4.8	3.7	6.5	4.0	2.7	2.5
oleic acid (C18:1)	23.3	24.4	63.9	39.7	36.7	39.6	26.1	28.8
linoleic acid (C18:2)	55.9	54.3	-	11.7	27.7	19.1	53.4	54.1
saturated chains	20.7	14.3	36.1	37.5	35.6	37.1	20.5	15.1
unsaturated chains	79.2	78.7	63.9	51.4	64.4	58.7	79.5	82.9

a Values determined by GC-MS using relative distribution.

Remarkably, our photocatalytic system enabled efficient hydrodecarboxylation of highly unsaturated feedstocks, achieving conversions above 90% for palm oil (PO), distilled crude oil (DCO), and waste cooking oil (WCO). This performance highlights both the robustness and the broad applicability of the method, especially considering the complexity and high unsaturation levels of real lipid biomass.

In contrast, soybean oil (SO), despite having a similar degree of unsaturation to DCO as confirmed by crude mixture chromatograms (Figure 3), showed a significantly lower conversion (38%). We tentatively attribute this discrepancy to minor components in SO, such as phospholipids, sterols, or oxidation products, which are known to influence photocatalytic processes by interfering with light absorption, catalyst stability, or mass transfer. While detailed control experiments (e.g., oil pretreatment or phospholipid removal) were beyond the scope of the present proof-of-concept study, these results clearly indicate that the composition of minor components can play a decisive role in photocatalytic upgrading of complex feedstocks.

**Figure 3:**
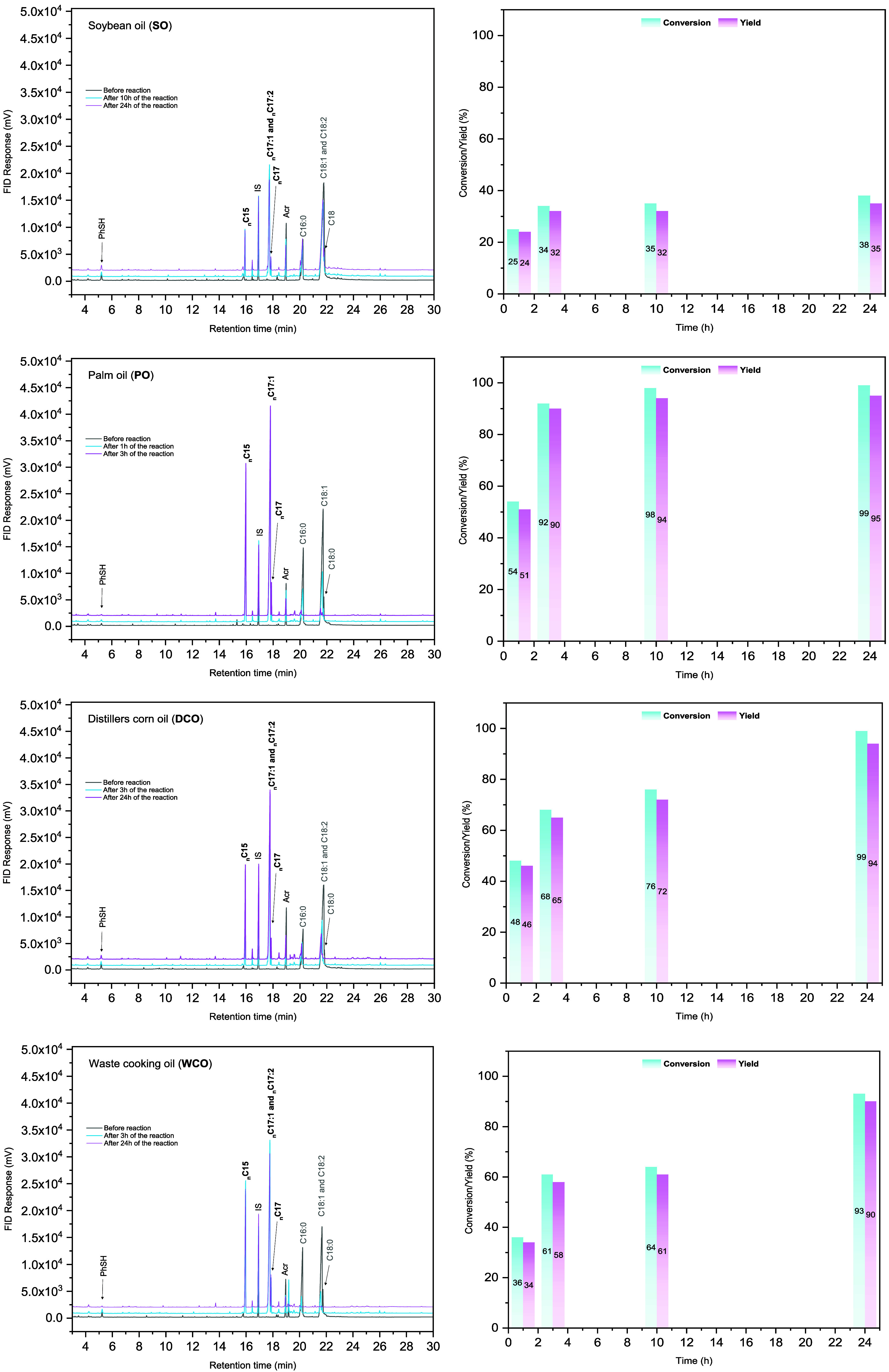
Chromatograms of bioderived fatty acids from SO, PO, WCO, and DCO before hydrodecarboxylation (gray) and after hydrodecarboxylation (blue and pink). Fatty acids: (C16:0) palmitic acid, (C18:0) stearic acid, (C18:1) oleic acid, (C18:2) linoleic acid; hydrocarbon products: (nC15) *n*-pentadecane, (nC17) *n*-heptadecane, (nC17:1) 8-heptadecene, (nC17:2) 6,9-heptadecadiene; (IS) internal standard. Inset graphs show the time-course of conversion and yield for each oil, illustrating fatty acid consumption and hydrocarbon formation over the reaction period.

Additional reaction time evaluation (Section 5.7, SI) revealed that, among the tested biomass-derived fatty acid mixtures, palm oil (PO) enabled significantly faster conversion, achieving a 90% yield in just 3h of visible light irradiation. This highlights PO as a particularly suitable raw material for this methodology. Under these conditions, the photocatalytic system afforded a turnover number (TON) of 8.28 and a turnover frequency (TOF) of 2.76 h^–1^.

The remarkable compatibility of the acridine/thiophenol system with highly unsaturated substrates can be attributed to its unique electronic and mechanistic properties. Acridine, a neutral aromatic nitrogen heterocycle, exhibits a lower excited-state reduction potential than typical acridinium salts, minimizing overoxidation and side reactions commonly observed with polyunsaturated substrates.^38,39^ This favorable electronic profile, combined with acridine’s inherent stability and its ability to perform proton-coupled electron transfer (PCET) under mild conditions, enables selective C–C bond cleavage with reduced impact on sensitive C=C bonds. These features may help explain the high conversions and selectivities observed for linoleic acid and complex real feedstocks, such as DCO and WCO, which have traditionally posed challenges in photocatalytic hydrodecarboxylation.

To assess the environmental impact and sustainability of the process, we calculated the E-factor following Sheldon’s definition, considering the mass of unrecycled inputs and excluding the solvent (recycled during the process) and gaseous CO_2_ (expected byproduct). Using 0.4 mmol of palm-oil-derived fatty acids (based on an average molar mass of 274.4 g/mol), the system achieved 99% conversion and 95% yield after 10 h. Under these conditions, an E-factor of 0.18 was obtained, reflecting intrinsic efficiency and low waste generation even in batch operation.

Scalability was demonstrated by increasing the substrate scale from 0.4 mmol to 1 g of palm oil fatty acids. Although scaling up extended the reaction time from 3 to 24 h, mainly due to photon penetration and mixing limitations inherent to batch reactors, the method consistently delivered excellent conversion (99%) and yield (95%). Importantly, energy consumption per mole of product decreased from 558 to 491 kWh/mol, underscoring the energy efficiency advantages of a larger-scale operation. These encouraging results validate the practical applicability of the method and suggest the potential for further improvements, such as employing continuous-flow photoreactors or optimizing light delivery to reduce reaction time and energy demand, thereby enabling sustainable industrial biofuel production.

The hydrocarbon mixtures obtained via photocatalytic hydrodecarboxylation of the studied oils are mainly composed of *n*-pentadecane (C15), *n*-heptadecane (C17), 8-heptadecene (C17:1), and 6,9-heptadecadiene (C17:2). The proportion of these hydrocarbons closely follows the initial fatty acid composition of each oil, and the overall selectivity for the deoxygenation of the oils is approximately 95%, in line with the results obtained for isolated fatty acids. In addition, no evidence of isomerization was observed under the reaction conditions. The resulting hydrocarbons are chemically compatible with green diesel and aviation biokerosene, confirming their direct applicability as renewable fuel components.^40,41^

Specifically, the renewable hydrocarbon mixture obtained from waste oils (WCO and DCO), which cost 2–3 times less than vegetable oils, contributes to reducing the overall cost of biofuel production^42^ and emerges as a viable strategy to overcome current barriers in the generation of advanced drop-in biofuels.^43^ To fully realize this potential, however, it is essential to develop catalytic systems that are not only effective but also compatible with the inherent complexity of real lipid feedstocks.

Although thermocatalytic technologies have demonstrated good performance in biomass deoxygenation, most studies focus on isolated fatty acids, with few examples progressing to real mixtures derived from vegetable oils or industrial lipid waste as feedstocks.^44^ These approaches, while effective, often require high temperatures (>200 °C), pressurized hydrogen gas, and metal-based catalysts, frequently involving noble metals, which must be presynthesized to display deoxygenation activity. Additionally, product selectivity tends to be low due to competing pathways (e.g., hydrodeoxygenation, decarboxylation, and cracking) induced by harsh conditions (Table 4)

**Table 4:** Overview of Thermocatalytic Approaches for Converting Fatty Acids into Renewable Hydrocarbons

feedstock	catalyst	solvent	temp (°C)	Atm	product	yield (%)	refs
palm fatty acid	NiCo/SBA-15-NH_2_	free	300	under N_2_	C_*n*–1_, C_*n*_H_2*n*+2_ and cracked hydrocarbons	83	^45^
distillate (PFAD)	Co/AC	free	350	under N_2_	C_*n*–1_, C_*n*_H_2*n*+2_ and cracked hydrocarbons	38	^46^
industrial FFA waste	Ni–Cu/Al_2_O_3_	dodecane	375	under H_2_	C_*n*–1_, C_*n*_H_2*n*+2_ and cracked hydrocarbons	>95%	^47^
tall oil fatty acid (TOFA)	Pd/C	dodecane	350	H_2_, 17 bar	C_*n*–1_ hydrocarbons	32	^48^
	Ni/γ-Al_2_O_3_	dodecane	300	H_2_, 30 bar	C_*n*_H_2*n*+2_ hydrocarbons	90	^49^
crude jatropha FFA	W/Pt/TiO_2_	dodecane	360	N_2_, 40 bar	C_*n*–1_, C_*n*_H_2*n*+2_ hydrocarbons	90	^47^
fatty acids isolated	Pt/Nb_2_O_5_	dodecane	180–250	H_2_, 8 bar	C_*n*_H_2*n*+2_ hydrocarbons	89–99	^50^
SO (FFA)	Acr/PhSH	DCM/H_2_O	rt	under N_2_ or open to air	C_*n*–1_ hydrocarbons	35	this work
PO (FFA)						95	
WCO (FFA)						95	
DCO (FFA)						90	

In contrast, our method operates under visible light, room temperature, atmospheric pressure, N_2_ or open-air, and hydrogen-free conditions, directly converting real fatty acid mixtures to C_*n*–1_ hydrocarbons with excellent selectivity. The operationally simple system uses low-cost, commercially available catalysts with a high practical applicability.

A potential limitation is the use of DCM, a nonrenewable solvent unsuitable for direct fuel use. However, thanks to the mild conditions of the process, DCM can be easily removed, due to its low boiling point, and efficiently recovered for reuse, minimizing both environmental and economic impact.

To the best of our knowledge, this is the first report of an organophotocatalytic system operating under mild conditions and directly applied to complex, lipid-based feedstocks for the production of hydrocarbons suitable as both drop-in and advanced biofuels.^51,52^ The combination of catalytic simplicity and compatibility with real biomass inputs represents a meaningful advancement in the development of sustainable methods for industrial biofuel production.

## 3 Conclusions

In summary, we have developed a dual photocatalysis system using acridine/thiophenol, composed of inexpensive and commercially available reagents, for the production of renewable C_*n*–1_ hydrocarbons via fatty acid hydrodecarboxylation. The methodology operates under ambient temperature and pressure, without requiring hydrogen gas, and effectively deoxygenates a broad scope of isolated saturated and unsaturated fatty acids as well as a controlled mixture of lauric and myristic acids, with yields generally exceeding 70%. We applied the method to hydrolyzed fatty acids from soybean oil, palm oil, waste cooking oil, and distillery corn oil, producing hydrocarbon mixtures compatible with green diesel and aviation biokerosene. Importantly, the reaction was also successfully scaled to 1 g of oil, achieving quantitative conversion and demonstrating the robustness of the system on a practical scale. The process exhibits an excellent green chemistry profile with an E-factor of 0.18. Overall, our methodology provides an efficient and sustainable route to renewable hydrocarbons from waste oils, enabling the production of potential advanced drop-in biofuels. We envision future work focusing on the exploration of alternative solvents and further scale-up using continuous-flow reactors to enhance the industrial applicability.

## 4 Experimental Section

### 4.1 General Procedure for the Hydrodecarboxylation of Fatty Acids

Fatty acid (1.0 equiv, 0.4 mmol), acridine (0.1 equiv, 0.04 mmol), and thiophenol (0.2 equiv, 0.08 mmol) were added to a 10 mL glass vial that had been previously dried in an oven and equipped with a magnetic stirring bar. The vial was then sealed, evacuated under pressure, and purged with nitrogen gas. A mixture of dichloromethane (DCM) and water (1.0 mL, 9:1) was added. The reaction mixture was irradiated with a 70 W violet LED (405 nm) under ventilation and stirred for 3–10 h.

For the hydrodecarboxylation of the real mixture of fatty acids obtained by hydrolysis of vegetable oils, 100 mg of starting material (≈0.4 mmol based on the average molar mass of the fatty acid mixture) was used, and the amount of catalysts was determined for a 0.4 mmol scale following the general procedure. The gram-scale hydrodecarboxylation of palm oil fatty acids was carried out using the same procedure with 1 g of starting material.

### 4.2 Determination of Product Conversion and Yield by GC-FID and GC-MS

The products were detected by GC-FID (Shimadzu, GC 2010) using a DB-5 column and quantified using a calibration curve of the hydrocarbon standard DRH-008S-R2 from Accustandard. The unsaturated hydrocarbons were quantified by using hexadecane as an internal standard and identified by GC-MS (Shimadzu, GCMS-QP2020) using an RTX-5MS column. The conversion and yield of each component were calculated using eqs 1, ([Disp-formula eq2]), and ([Disp-formula eq3]). The conversion and yield of each component were calculated using the following eqs 1, ([Disp-formula eq2]), and ([Disp-formula eq3])­

1 $$c\mathrm{onversion}\left[\%\right]=\left(1-C_{F}/C_{F}^{0}\right)\times 100\%$$

where *C*_F_ and *C*_F_^0^ are the contents of fatty acids in the product and reactant, respectively.

2 $$y\mathrm{ield}\left[\%\right]=C_{H}/C_{\mathrm{Hmax}}\times 100\%$$

where *C*_H_ and *C*_Hmax_ are the hydrocarbon contents in the product and the maximum theoretical content, respectively.

The conversion of fatty acids from vegetable oils was determined using the sum of peak areas of the reactants before and after the reaction according to eq 3

3 $$c\mathrm{onversion}\left[\%\right]=\left(1-\sum A/\sum A^{0}\right)\times 100\%$$

where *A* and *A* ^0^ are the peak areas of each component of the oils at beginning and at the end of the reaction.

## Supplementary Material



## Notes

General procedure for the hydrodecarboxylation of fatty acids; reaction layout; solvent screening; additives screening; optimization of catalyst stoichiometry; control experiments; optimization of time for hydrodecarboxylation of hydrolyzed vegetable oils; cost comparison between similar photocatalytic systems; reagent cost comparison (price per mol) (PDF)
