# Supplementary material for: Dual Acridine/Thiophenol Photocatalysis for the Production of Advanced Drop-in Biofuels
Source: ACS Omega. 2025 Sep 25;10(39):45992–6001. doi: 10.1021/acsomega.5c06908 (PMC12508946; doi:10.1021/acsomega.5c06908)
Supplement: Supplementary file 1 [file ao5c06908_si_001.pdf]

SUPPORTING INFORMATION

DUAL ACRIDINE/THIOPHENOL PHOTOCATALYSIS FOR THE PRODUCTION OF ADVANCED DROP-IN BIOFUELS

Jhudson Guilherme Leandro de Araujo[a]
Jannyely Moreira Neri[a]
Aruzza Mabel de Morais Araújo[a]
Carlos Alberto Martínez-Huitle[a]
Amanda Duarte Gondim[a]
Lívia Nunes Cavalcanti[a]*

[a]Federal University of Rio Grande do Norte, Institute of Chemistry, Campus Universitário, Lagoa Nova, Natal, RN 59072-970, Brazil.

Corresponding author. E-mail:livia.cavalcanti@ufrn.br*

Table of Contents

1. **General Information** S3
2. **General procedure for the hydrodecarboxylation of fatty acids** S3
3. **Procedure for vegetable oils hydrolysis** S4
4. **Determination of Product Conversion and Yield by GC-FID and GC-MS** S4
5. **Optimization of the Reaction Conditions** S5
6. **Price of Reagents and cost comparison** S9
7. **Supplementary References** S9

1. General Information

All reagents and solvents were purchased (Sigma Aldrich, Dinâmica, Neon, Synth, Vetec) and used as received without further purification. Chromatography with Flame Ionization Detection (GC-FID) analyses were performed using a Shimadzu GC 2010 instrument equipped with a DB-5 column (0.25 mm x 30 m, Film: 0.25 µm, Agilent). Gas chromatography mass spectra (GC-MS) were taken at Thermo Scientific ISQ Quadrupole GC-MS with Trace GC Ultra equipped with a DB-5MS column (0.25 mm x 30 m, Film: 0.50 µm, Agilent).

2. General procedure for the hydrodecarboxylation of fatty acids

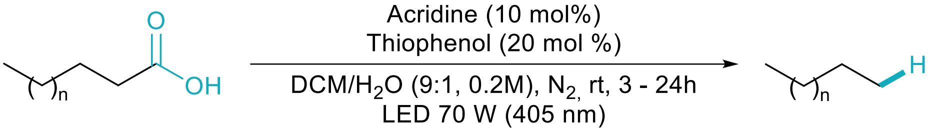

Fatty acid (1.0 eq., 0.4 mmol), acridine (0.1 eq., 0.04 mmol), and thiophenol (0.2 eq., 0.08 mmol) were added to a 10 mL glass vial that had been previously dried in an oven and equipped with a magnetic stirring bar. The vial was then sealed, evacuated under pressure, and purged with nitrogen gas. A mixture of dichloromethane and water (1.0 mL, 9:1) was added. The reaction mixture was irradiated with a 70 W violet LED (405 nm) under ventilation and stirred for 3 - 10 h.

For the hydrodecarboxylation of the real mixture of fatty acids obtained by hydrolysis of vegetable oils, 100 mg of starting material (~0.4 mmol based on the average molar mass of the fatty acid mixture) was used, and the amount of catalysts was determined for a 0.4 mmol scale following the general procedure. The gram-scale hydrodecarboxylation of palm oil fatty acids was carried out using the same procedure with 1 g of starting material.

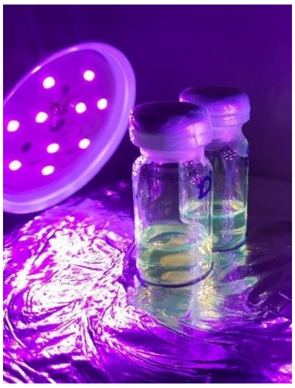

Figure S1 – Reaction layout

3. Procedure for vegetable oils hydrolysis

The free fatty acids were obtained by adapting the procedure described in the literature.[1] First, 5.0 g of vegetable oil and 30 mL of ethanolic KOH saponifying solution (90%) (1.75 M) were mixed in a round-bottom flask. The reaction was heated to 65°C under vigorous agitation until the starting material was consumed. Monitoring was done by thin layer chromatography (TLC). After the hydrolysis, 30 mL of water was added to the mixture, and the unsaponifiables were separated by extraction with 2 x 20 mL of hexane. The alcoholic phase containing the soaps was acidified to pH 1 with HCl 6 M, and the fatty acids were recovered by extraction with hexane. The extract was washed with distilled water until neutral pH was achieved. The hexane fraction was dried with anhydrous magnesium sulfate, and the solvent was rotary evaporated to obtain 3.45 g of fatty acids.

4. Determination of Product Conversion and Yield by GC-FID and GC-MS

The products were detected by GC-FID (Shimadzu, GC 2010) using a DB-5 column and quantified using a calibration curve of the hydrocarbon standard DRH-008S-R2 from Accustandard. The unsaturated hydrocarbons were quantified using hexadecane as an internal standard and identified by GC-MS (Shimadzu, GCMS-QP2020) using an RTX-5MS column. The conversion and yield of each component were calculated using Equations (1), (2), and (3). The conversion and yield of each component were calculated using the following Equations (1), (2), and (3):

$$\text{Conversion [\%]} = \left(1 - \frac{C_F}{C_F^0}\right) \text{ x } 100\% \qquad (1)$$

where C_F and C_F^0 are the contents of fatty acids in the product and reactant, respectively.

$$\text{Yield [\%]} = \frac{C_H}{C_{Hmax}} \text{ x } 100\% \qquad (2)$$

where C_H and C_{Hmax} are the hydrocarbon contents in the product and the maximum theoretical content, respectively.

The conversion of fatty acids from vegetable oils was determined using the sum of peak areas of the reactants before and after the reaction according to equation (3):

$$\text{Conversion [\%]} = \left(1 - \frac{\sum A}{\sum A^0}\right) \text{x } 100\% \qquad (3)$$

where A and A° are the peak areas of each component of the oils at beginning and at the end of the reaction.

5. Optimization of the Reaction Conditions

Table S1.1 – Solvent Screening[a]

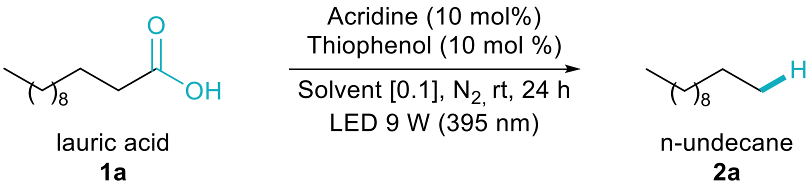

Entry	Solvent	Yield (%)[b]
1	DCM	16
2	MeCN	<5
3	Tolueno	19
4	AcOEt	23
5	DCE	8
6	MeOH	0
7	THF	<5
8	DMA	0
9	DMF	0
10	H_2O	11
11	DCM/H_2O (9:1)	**37**
12	Tolueno/H_2O (9:1)	16
13	AcOEt/H_2O (9:1)	15
14	DCE/H_2O (9:1)	11
15	MeOH/H_2O (9:1)	<5
16	DCM/H_2O (4:1)	16
17	DCM/H_2O (19:1)	19

[a]Reaction conditions: fatty acid 1a (0.4 mmol), acridine (10 mol%), thiophenol (10 mol%), Solvent (0.1 M), 9W LED (395 nm), N_2, rt, 24h. [b]Determined by GC-FID using a calibration curve

Table S1.2 – Additives screening[a]

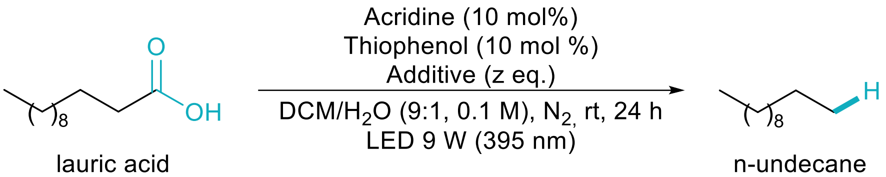

lauric acid **1a** — n-undecane **2a**

Entry	Additive (z eq.)	Yield (%)[b]
1	DIPEA (0.2)	16
2	Piridina (0.2)	<5
3	K_2CO_3 (0.2)	19
4	K_2HPO_4 (0.2)	23
5	KH_2PO_4 (0.2)	8
6	Na_2HPO_4. $12H_2O$ (0.2)	0
7	NaH_2PO_4. H_2O (0.2)	<5
8	$NaBO_3{\cdot}_nH_2O$ (0.5)	0
9	$LiClO_4$ (0.5)	11
10	$K_2S_2O_8$ (0.5)	37
11	$NaIO_4$ (0.5)	16
12	$NaIO_3$ (0.5)	15
13	$KHSO_5$ (0.5)	11
14	H_2O_2 (0.5)	
15	DIPEA (0.5)	19
16	KCl (20)	28
17	KBr (20)	31
18	KI (20)	24

[a]Reaction conditions: fatty acid 1a (0.4 mmol), acridine (10 mol%), thiophenol (10 mol%), DCM:H_2O (9:1, 0.1 M), 9W LED (395 nm), N_2, rt, 24h. [b]Determined by GC-FID using a calibration curve.

Table S1.3 – Optimization of catalyst stoichiometry[a]

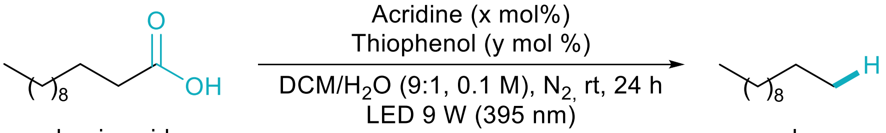

lauric acid **1a** — n-undecane **2a**

Entry	Acridine (x mol %)	Tiofenol (y mol%)	Yield (%)[b]
1	100	100	76
2	10	100	69
3	10	40	62
4	10	30	62
5	10	20	**69**
6	10	5	<5
7	100	10	67

8	40	10	69
9	30	10	69
10	20	10	68
11	5	10	<5
12	1	10	<5
13	5	20	17
14	5	5	<5
15	20	5	26
16	20	20	35
17	5	10	22

[a]Reaction conditions: fatty acid 1a (0.4 mmol), DCM:H_2O (9:1, 0.1 M), 9W LED (395 nm), N_2, rt, 24h. [b]Determined by GC-FID using a calibration curve.

Table S1.4 – Optimization of concentration[a]

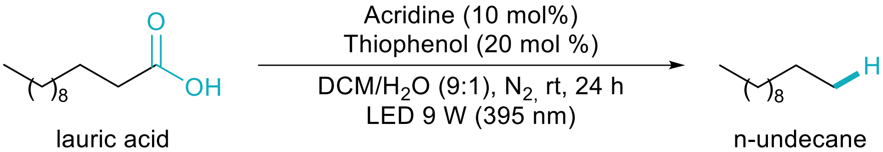

lauric acid **1a** | n-undecane **2a**

Entry	V (mL)	[1a]	Yield (%)[b]
1	4.0	0.05	22
2	1.0	0.2	**72**
3	0.5	0.4	28

[a]Reaction conditions: fatty acid 1a (0.4 mmol), acridine (10 mol%), thiophenol (20 mol%), DCM:H_2O (9:1), 9W LED (395 nm), N_2, rt, 24h. [b]Determined by GC-FID using a calibration curve.

Table S1.5 – Optimization of LED and reaction time[a]

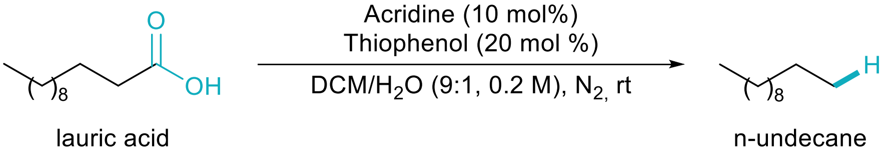

lauric acid **1a** | n-undecane **2a**

Entry	LED	Time	Yield[b]
1	70 W violet (405 nm)	24h	71
2	7 W blue (~ 450 nm)	24h	0
3	9 W violet (395 nm)	1h	15
4	9 W violet (395 nm)	3h	26
5	9 W violet (395 nm)	6h	50
6	9 W violet (395 nm)	9h	69
7	9 W violet (395 nm)	12h	70
8	70 W violet (405 nm)	1h	55

9	70 W violet (405 nm)	3h	**72**
10	70 W violet (405 nm)	6h	65
11	70 W violet (405 nm)	9h	68
12	70 W violet (405 nm)	12h	66

[a]Reaction conditions: fatty acid 1a (0.4 mmol), DCM:H_2O (9:1, 0.2 M), N_2, rt. [b]Determined by GC-FID using a calibration curve.

Table S1.6 – Control experiments[a]

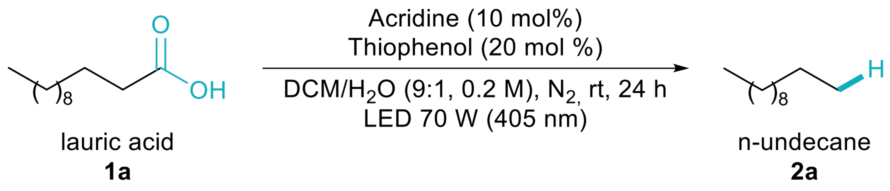

Entry	Control conditions	Yield (%)[b]
1	no thiophenol	<5
2	no acridine	0
3	no LED	0

[a]Reaction conditions: fatty acid 1a (0.4 mmol), acridine (10 mol%), thiophenol (20 mol%), DCM:H_2O (9:1, 0.2 M), 70W LED (405 nm), N_2, rt, 24h. [b]Determined by GC-FID using a calibration curve.

Table S1.7 – Optimization of Time for hydrodecarboxylation of hydrolyzed vegetable oils [a]

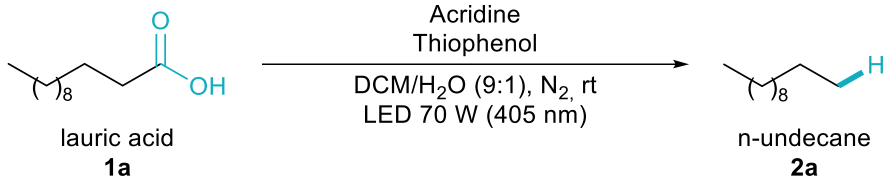

	Hydrolyzed oil	Conversion x Time			
		1h	3h	10h	24h
1	Soybean oil (SO)	25%	34%	35%	38%
2	Palm oil (PO)	54%	92%	98%	99%
3	Waste cooking oil (WCO)	48%	68%	76%	99%
4	Distillery corn oil (DCO)	36%	61%	64%	93%

[a]Reaction conditions: mix fatty acid (100 mg), acridine (0.04 mmol), thiophenol (0.08 mmol), DCM:H_2O (9:1) (2 mL), N_2, rt. [b]Determined by GC-FID using a calibration curve.

6. Price of Reagents and cost comparison

Table 2.1 – Cost comparison between similar photocatalytic systems

Reagents	This work	Li's group work[2]
Acridine	10 mol %	96,78
Thiofenol	20 mol %	9,52
Mes-1,3,6,8-tetramethoxy-Acr-3",5"-dimethoxy-Ph	10349,8	2.5 mol %
P-Tolyl disulfide	11,58	5 mol %
Tetrabutylammonium acetate	2,41	2.5 mol %
**Cost/mol ($)**	10363, 8	106.3

Table 2.2 – Reagent cost comparison (price per mol)

**Reagents**	**CAS No.**	**Mw/Conc.**	**Vendor**	**Price listed ($)**	**Price/mol($)**
Acridine	260-94-6	179.22	combi-blocks	540/100g	967.8
Thiofenol	108-98-5	110.18	oakwoodchemical	1080/2.5kg	47.6
Mes-1,3,6,8-tetramethoxy-Acr-3",5"-dimethoxy-Ph	1965330-59-9	641.46	ambeed	645/1g	413992.3
P-Tolyl disulfide	103-19-5	246.4	combi-blocks	470/500g	231,6
Tetrabutylammonium acetate	10534-59-5	301.51	combi-blocks	320/1kg	96.5

7. Supplementary References

(1) Salimon, J.; Abdullah, B. M.; Salih, N. Hydrolysis Optimization and Characterization Study of Preparing Fatty Acids from Jatropha Curcas Seed Oil. *Chem. Cent. J.* **2011**, *5* (1), 1–9. https://doi.org/10.1186/1752-153X-5-67.

(2) Sun, Y. L.; Tan, F. F.; Hu, R. G.; Hu, C. H.; Li, Y. Visible-Light Photoredox-Catalyzed Hydrodecarboxylation and Deuterodecarboxylation of Fatty Acids. *Chinese J. Chem.* **2022**, *40* (16), 1903–1908. https://doi.org/10.1002/cjoc.202200143.
